# Assessment of grip-motion characteristics in carpal tunnel syndrome patients using a novel finger grip dynamometer system

**DOI:** 10.1186/s13018-020-01773-9

**Published:** 2020-07-06

**Authors:** Toru Sasaki, Koji Makino, Akimoto Nimura, Shiro Suzuki, Tomoyuki Kuroiwa, Takafumi Koyama, Atsushi Okawa, Hidetsugu Terada, Koji Fujita

**Affiliations:** 1 Department of Orthopaedic and Spinal Surgery, Graduate School of Medical and Dental Sciences, Tokyo Medical and Dental University, 1-5-45, Yushima, Bunkyo-ku, Tokyo, 113-8519 Japan; 2 Center for Creative Technology, University of Yamanashi, 4-3-11, Takeda, Kofu, Yamanashi, Japan; 3 Department of Functional Joint Anatomy, Graduate School of Medical and Dental Sciences, Tokyo Medical and Dental University, 1-5-45, Yushima, Bunkyo-ku, Tokyo, 113-8519 Japan; 4 Department of Mechatronics, University of Yamanashi, 4-3-11, Takeda, Kofu, Yamanashi, Japan

**Keywords:** Carpal tunnel syndrome, Grip dynamometer, Grip motion, Principal component analysis

## Abstract

**Background:**

Grip strength measurement is widely used in daily medical practice, and it has been reported that the grip strength decreases in patients with carpal tunnel syndrome (CTS). However, conventional grip dynamometers evaluate only the maximum power of total grip strength and cannot measure the time course of grip motion. In this report, we aimed to determine the grip characteristics of CTS patients by measuring the time course of each finger’s grip motion and to analyze the relationship between finger grip strength and subjective symptoms using this new grip system.

**Methods:**

The grip strength of each finger was measured using the new grip system that has four pressure sensors on the grip parts of each finger of the Smedley grip dynamometer. We analyzed the time course of grip motion and relationship between finger grip strength and subjective symptoms in 104 volunteer and 51 CTS hands. The Japanese Society for Surgery of the Hand version of the Carpal Tunnel Syndrome Instrument (CTSI-JSSH) and the Disability of Arm, Shoulder, and Hand questionnaire (DASH) were used as subjective evaluation scores.

**Results:**

In the CTS group, the grip time with the index, middle, and ring fingers was longer, and the time at which strength was lost after reaching the maximum was earlier. Patients with severe subjective symptoms tended to not use the index and middle fingers during grip motion.

**Conclusions:**

This new system that measures each finger’s grip strength at one time and record the time course of grip motion could quantify a patient’s symptoms easily and objectively, which may contribute to the evaluation of hand function.

## Background

Grip strength measurement is widely used in daily medical practice because it is a non-invasive, quick, and inexpensive way of assessing muscle strength. In the field of hand surgery, grip strength may decrease due to pain and neuropathy; thus, it is important to measure the grip strength to evaluate hand function [1–3]. In particular, it has been shown that grip strength is decreased in patients with carpal tunnel syndrome (CTS) [4, 5], which is a clinical syndrome of numbness, pain, and disorder of thumb opposition associated with localized compression of the median nerve at the wrist [6–9]. However, the grip characteristics of patients with CTS are still unclear, and the mechanism underlying diminished grip strength in those with damaged sensory and motor fibers of the median nerve needs to be investigated.

A conventional grip dynamometer, which has been used in many studies that have demonstrated weakened grip strength in patients with CTS, evaluates only the maximum power in the total grip strength of all fingers. Moreover, there have been no reports about the time course of grip motion, such as the time from the beginning of grip motion to maximum power and the time from maximum power to loss of grip strength. Thus, it was necessary to develop a grip dynamometer that can measure the time course to clarify the characteristics of grip motion in CTS patients.

Although the Japanese Society for Surgery of the Hand version of the Carpal Tunnel Syndrome Instrument (CTSI-JSSH) and the Disability of Arm, Shoulder, and Hand questionnaire (DASH) are used as subjective evaluation scores in CTS patients, and the usefulness of these scores as indicators of patient’s symptom has been reported [10], there is no report on the correlation between subjective symptoms of CTS and grip strength. To evaluate the subjective symptoms of patients with only distal median nerve dysfunction using a grip dynamometer, we needed a new system for measuring the grip strength of each finger, since it is difficult to accurately evaluate these symptoms using the total grip strength of all fingers.

Therefore, we developed a new grip system that could measure the grip strength of each finger in a single instance to record the time course and analyze the grip motion of healthy volunteers [11]. In this report, to understand the pathophysiology of CTS, we aimed to analyze the grip characteristics of CTS patients by measuring the time course of the grip motion, and we sought to determine the relationship between finger grip strength and subjective symptoms using this new grip system.

## Methods

This study was approved by the institutional review board of our institution, and we obtained informed consent from all patients.

### Participants

We included 51 hands of 30 patients with CTS (bilateral CTS—21, unilateral CTS—9) who were diagnosed by hand surgeons (CTS group) and 104 hands of 52 healthy volunteers (control group). The inclusion criteria for this study were as follows: clinical symptoms of CTS (numbness, tingling, and pain), positive examination for CTS including a positive Phalen’s sign and Tinel’s sign, and an abnormal nerve conduction study (NCS) with sensory nerve conduction velocity (SCV) of ≤ 44 m/s or abductor pollicis brevis–distal latency (APB-DL) of > 4.0 ms. Patients who continued to have symptoms and an abnormal NCS after carpal tunnel release were included. Patients were excluded from this study if they had compressive neuropathy in the ipsilateral arm or peripheral polyneuropathy, cervical disease, DeQuervein syndrome, or trigger finger, had a history of a distal radial fracture, were pregnant, and were non-Japanese speaking. The NCS results were classified according to the Bland classification, which is based on the electrophysiological severity [12].

For the control group, the inclusion criteria included participants who had no symptoms or previous history of neuropathies or trauma to the upper limbs, and they were age-matched to the participants in the CTS group.

### Measurement

**Fig. 1:**
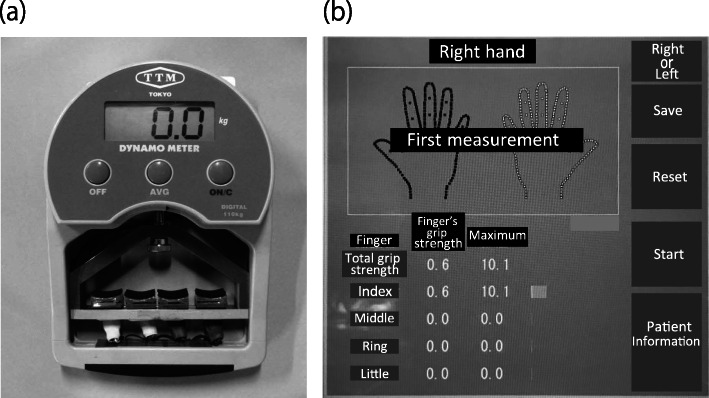
The finger grip dynamometer system. **a** Measurement unit: four pressure sensors on the grip parts of each finger (index, middle, ring, and little fingers) of the Smedley grip dynamometer. The four sensors measure the voltage values and send the data to the display through a wireless communication unit. **b** Display unit: the display converts measuring date to grip strength and shows date in real time

The grip strength of each finger was measured using the new grip system developed in our institution. The apparatus used and measurement posture were the same as those in the previous report, and data regarding the healthy volunteers have already been reported [11]. The structure of this system was the same as that in the previous report and is shown in Fig. 1. This system consists of three units, which are as follows: a measurement unit with four pressure sensors on the grip parts of each finger (index, middle, ring, and little fingers) of the Smedley grip dynamometer (Fig. 1a), a wireless communication unit that sends the data to the display every 10 ms, and a display unit that shows each finger’s grip strength in real time (Fig. 1b). When the patients grip this system, the four sensors measure the voltage values and send the data to the display through a wireless communication unit. The voltage values are converted to grip strength, and each finger’s grip strength is displayed and saved on the computer in real time.

The right and left finger grip strengths were measured twice [13, 14] using this system while the patient was standing with the shoulders adducted, elbows in the straight position, and forearms and wrists in the neutral position. The average of the two trials was calculated. The patients were instructed to hold the device as hard and as early as possible and to release it when the power reached the maximum.

### Statistical analysis

Data regarding age, CTSI-JSSH, DASH, and measured grip strength are presented as medians with interquartile ranges. The Student *t* test, Fisher exact test, and Mann-Whitney *U* tests were used to compare differences in nominal and nonparametric variables. Based on previous studies, we considered a 3.0-kg decrease in the grip strength as clinically meaningful when comparing the difference in grip strength between the CTS patients and controls [15, 16]. We calculated that, with a sample of 88 participants (44 participants per group), the study would have 80% power to detect a 3.0-kg mean decrease in the grip strength, with a type 1 error of 5%. For the power analysis, we used a standard deviation of 5.0 in the grip strength using data reported in previous studies [17]. To evaluate the characteristics of grip motion in the CTS patients, we performed the following types of analysis.

First, to compare the time course of finger grip strength between the CTS and control groups, the grip time at which the maximum grip strength was reached was analyzed. We calculated the time from reaching 20% of the maximum strength to reaching the maximum strength [18, 19]. We compared the grip time between the CTS and control groups using *t* tests. In addition, we analyzed the mechanism underlying the diminished grip strength in CTS patients. The ratio of the maximum finger grip strength to real time finger grip strength was considered to be the ΔGrip strength. We calculated ΔGrip strength from − 1.6 to + 3.2 s of the time when the maximum grip strength was reached. At each time point, the average of ΔGrip strength of the CTS and control groups was calculated and visualized graphically for each finger. Since the absolute values of each finger’s grip strength were not being compared, differences in gender and individual physique have less of an influence on the grip time and ΔGrip strength.

Second, to evaluate the relationship between subjective symptoms and finger grip strength in CTS patients, a principal component analysis (PCA) of each finger’s grip strength and subjective symptom scores was performed in the CTS group. PCA is a multivariate statistical technique applied to systematically reduce the number of dimensions [20–22]. This method extracts the important information from the data to represent these data as a set of new orthogonal variables called principal components and to display the pattern similarity between the observations and the variables as points in maps. PCA results show that factors with arrows pointing in the same direction have a tendency to be correlated, while factors with scattered arrows have little correlation. In the PCA, we set the grip strength of the individual’s largest finger (index, middle, ring, or little finger) to 1.0 and calculated the ratio of the other fingers to that of the largest finger. PCA of their finger grip strength ratios and subjective scores was performed. For this reason, our results compare of the ratio of forces per finger; thus, it was not affected by differences in individual physique. The subjective symptom score was obtained using the CTSI-JSSH symptom severity scale (CTSI-SS), CTSI-JSSH functional condition scale (CTSI-FS), and DASH. In order to investigate the characteristics of body size, we performed PCA on body mass index (BMI) and finger grip strength.

## Results

The participants’ demographic features are presented in Table 1.

**Table 1:** Characteristics of the participants in the CTS and control groups

	Control group (*N* = 52)	CTS group (*N* = 51) Pre-operation = 40 Post-operation = 11	*p* value
Sex			0.288*
Male	19	13	
Female	33	38	
Age	70 (58–79)	73 (61–78.3)	0.35**
Bland classification		Grade 0, 1 Grade 1, 7 Grade 2, 3 Grade 3, 17 Grade 4, 0 Grade 5, 14 Grade 6, 9	
CTSI-SS		22 (16–25)	
CTSI-FS		37.0 (27.5–37.5)	
DASH		37.1 (22.0–41.2)	

Data are presented as median (IQR). Statistical significance was determined by the Fisher exact test and Mann-Whitney *U* test.

*IQR* interquartile range, *CTS* carpal tunnel syndrome,

*CTSI-SS* Japanese Society for Surgery of the Hand version of Carpal Tunnel Syndrome Instrument Symptom Severity Scale, *CTSI-FS* Japanese Society for Surgery of the Hand version of Carpal Tunnel Syndrome Instrument Functional Condition Scale, *DASH* Disabilities of the Arm Shoulder and Hand, *BMI* body mass index

*Fisher’s exact test

**Mann-Whitney *U* tests

### Time course of each finger’s grip strength

**Fig. 2:**
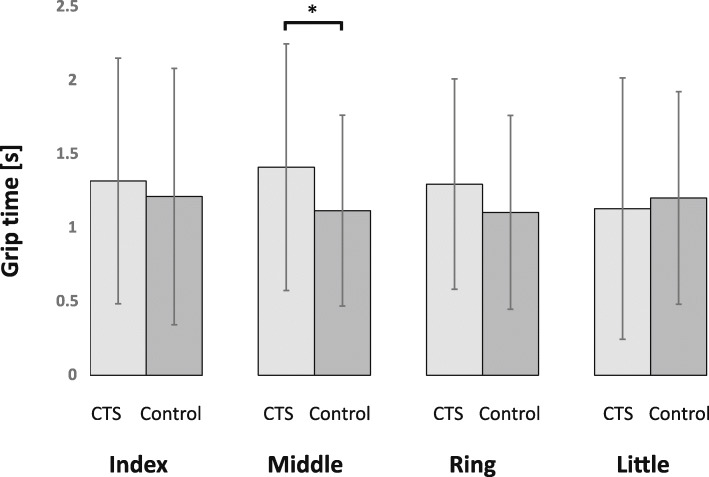
Grip time of each finger’s grip strength. Grip time with index, middle, and ring fingers is longer in the CTS patients than in the controls. Statistical significance was determined by using the Student *t* test. In the grip time with the middle finger, a significant difference is observed between the CTS patients and controls (*P* = 0.01)

Grip time with the index, middle, and ring fingers was longer in the CTS group than in the control group (Fig. 2). However, there was no difference in grip time with the little finger between the two groups. In particular, a significant difference (*P* = 0.01) was observed in the grip time of the middle finger.

**Fig. 3:**
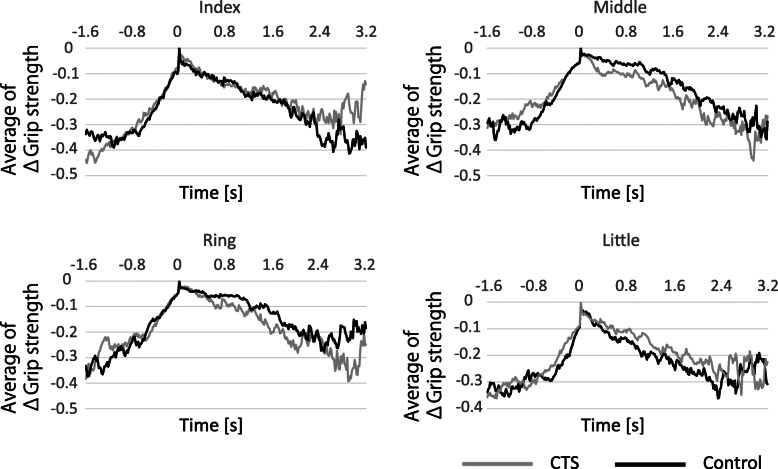
The time to loss of grip strength. The time at which grip strength was lost in the middle and ring fingers was earlier in the CTS patients than in the controls

Furthermore, in the time course of ΔGrip strength of the middle and ring fingers, the CTS group showed an early loss of strength after reaching the maximum grip strength than the control group (Fig. 3). In the time course of the little finger, the CTS group tended to release later than the control group. For the index finger, there was no difference between the CTS and control groups.

### Principal component analysis

**Fig. 4:**
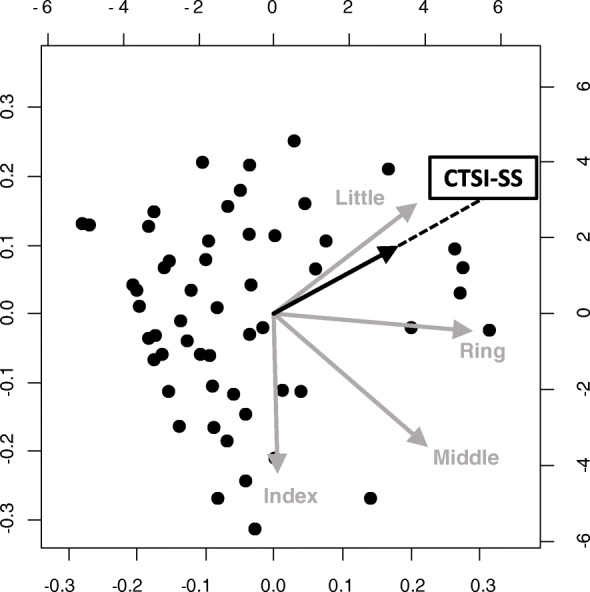
Result of the PCA of each finger’s grip strength and CTSI-SS

**Fig. 5:**
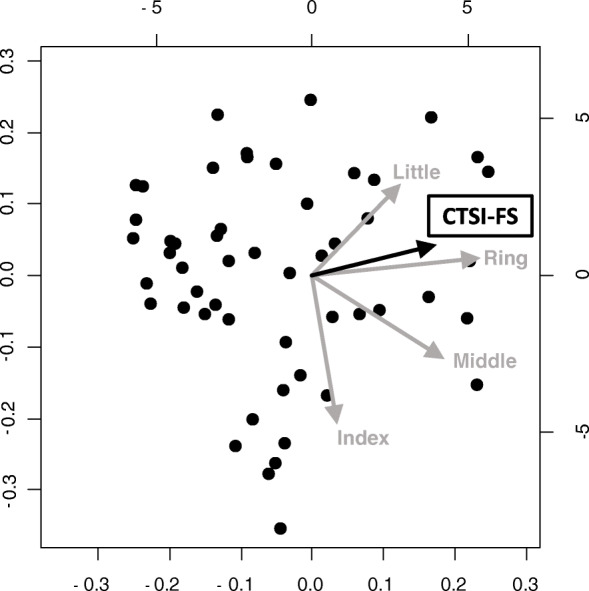
Result of the PCA of each finger’s grip strength and CTSI-FS

**Fig. 6:**
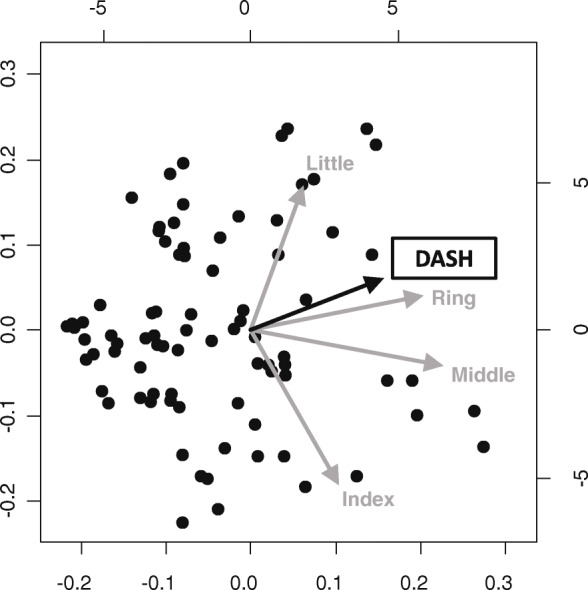
Result of the PCA of each finger’s grip strength and DASH

**Fig. 7:**
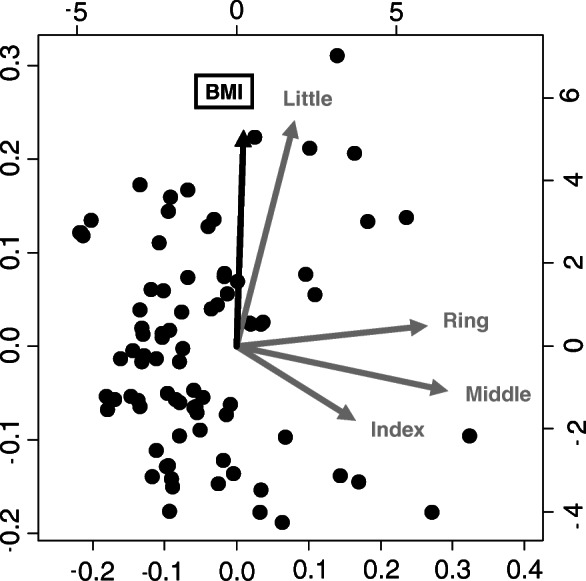
Result of the PCA of each finger’s grip strength and BMI

The results of the PCA of the subjective symptom score and each finger’s grip strength in the CTS group were examined. In the PCA of each finger’s grip strength and CTSI-SS, CTSI-SS was drawn between the ring and little fingers and showed a strong correlation with the ring finger (Fig. 4). In the PCA of each finger’s grip strength and CTSI-FS, CTSI-FS was drawn between the ring and little fingers and showed a strong correlation with the little finger, similar to the PCA of DASH (Figs. 5 and 6). In the PCA of each finger’s grip strength and BMI, the arrows of little finger grip strength and BMI pointed same direction (Fig. 7).

## Discussion

In this study, we analyzed the characteristics of grip motion in CTS patients and examined the relationship between grip strength and subjective symptoms (CTSI-JSSH and DASH) using a new grip system that can measure each finger’s grip strength at a single instance and record the time course of grip motion.

There have been no reports focused on the time course of grip motion, such as the time from the beginning of grip motion to the maximum power, and the time from maximum power to loss of grip strength. Our system could show the characteristics of CTS patients in whom the grip time of the index, middle, and ring fingers was long, and grip strength was lost early after it reached the maximum. In the little finger, the CTS group tended to release later than the control group. This may be due to the fact that the middle and ring fingers are released early in the CTS group and rely on the little finger for gripping. From these results, we may be able to quantitatively evaluate sensory and motor nerve disorders, which may affect the hand function of CTS patients.

Furthermore, a grip system was previously used to measure the maximum grip strength of each finger of healthy hands with anesthesia to the median nerve [23]. In that report, grip strength of the middle, ring, and little fingers decreased after anesthesia, which was not consistent with the pathophysiology of CTS; thus, this method, using anesthesia, was deemed inadequate to accurately evaluate the hand function of CTS patients. Conversely, in our study, the symptom severity correlated with the tendency of patients to not use the index and middle fingers during grip motion. These results were consistent with the pathophysiology of CTS.

A previous study using a conventional grip dynamometer reported that no correlation was found between grip strength and severity of CTS [24]. It was also demonstrated that grip motion requires a synergistic function of the intrinsic and extrinsic muscles of the hand and does not use the muscles affected by CTS [25]. Thus, the conventional grip dynamometer may not be accurate enough to evaluate the function of the hands with CTS. Conversely, in our PCA for CTSI-FS, CTSI-SS, DASH, and finger grip strength, the arrow was shown between the ring and little finger. In the PCA, factors with arrows pointing in the same direction have a tendency to be correlated. These results show that patients with severe subjective symptoms, as well as high CTSI-FS, CTSI-SS, and DASH scores, tended to not use the index and middle fingers during grip motion. These results proved that the function of the index and middle fingers worsens when the sensory nerve fibers of the digital nerve (index, middle, and ring fingers) and motor nerve fibers of the first and second lumbricals are damaged at the carpal tunnel. Thus, the finger grip dynamometer system could become a useful tool for evaluating the severity of CTS. It is not clear why patients with a high BMI are more likely to hold their little finger. In a study using a conventional grip strength dynamometer, people with a higher BMI were found to have greater grip strength [26]; thus, people with stronger grip strength may tend to grip on the ulnar side. We also analyzed the SCV and APB-DL of the NCS as an objective evaluation. Although PCA was performed on SCV, APB-DL, and finger grip strength, none showed a correlation between electrophysiological severity and finger grip strength (data not shown).

This study has several limitations. First, the CTS and control groups matched in age but not in sex. Although the comparison of the absolute value of the total grip strength and each finger’s grip strength is inadequate, the analysis of time course and the PCA were not evaluations of the absolute value of grip strength and were considered to be useful results. Second, because DASH is not a questionnaire that asked for answers on the left and right hand separately, DASH results do not necessarily reflect the subjective symptoms of hands measured by finger grip strength. CTSI is a questionnaire that asks for answers on the left and right hands separately.

## Conclusion

We developed a new grip system that can measure each finger’s grip strength at a single instance and record the time course of grip motion. Our system could show the characteristics of CTS patients in whom the grip time of the index, middle, and ring fingers was long, and grip strength was lost early after it reached the maximum. Furthermore, we could show that patients with severe subjective symptoms tended to not use the index and middle fingers during grip motion. From these results, our system could quantify the patient’s symptoms easily and objectively. To measure each finger’s grip strength in real time is an unprecedented method for characterizing the grip motion of patients with various diseases and understanding the pathophysiology of diseases. In the future, we would like to evaluate whether this finger grip dynamometer system is useful for diagnosis.

## Data Availability

The datasets used and/or analyzed during the current study are available from the corresponding author on reasonable request.

## Abbreviations

ABP-DL: Abductor pollicis brevis-distal latency
CTS: Carpal tunnel syndrome
CTSI-FS: CTSI-JSSH functional condition scale
CTSI-JHSS: Japanese Society for Surgery of the Hand version of the Carpal Tunnel Syndrome DASH Disability of Arm, Shoulder, and Hand questionnaire
CTSI-SS: CTSI-JSSH symptom severity scale
NCS: Nerve conduction study
PCA: Principal component analysis
SCV: Sensory conduction velocity
